# Potential Nutrient Contribution of Community-Based Insects in Children’s Food in Northern Ghana

**DOI:** 10.1016/j.cdnut.2024.104410

**Published:** 2024-07-02

**Authors:** Clement Kubreziga Kubuga, Majeed Baako, Jan W Low

**Affiliations:** 1 Nutritional Sciences Department, University for Development Studies, Tamale, Ghana; 2 International Potato Center, Nairobi, Kenya

**Keywords:** edible insects, entomophagy, community-based insects, food security, nutrition security

## Abstract

**Background:**

Micronutrient deficiencies are a major problem among children in northern Ghana. Available local foods and existing plant-based dietary patterns among children are insufficient to meet children’s nutrients requirements. Aside enhancing diets with animal source foods, most of which are expensive for rural households, entomophagy, which is culturally accepted, appears to be a great alternative.

**Objectives:**

This study aimed to *1*) document the types of insects commonly consumed and the reasons for or against entomophagy in the study area, *2*) document the reasons for adding or not adding insects to household meals, and *3*) determine the nutrient contribution of community-based insects in children's food.

**Methods:**

Both qualitative and quantitative research methods were concurrently applied in this exploratory study (*N* = 392 individuals; 6 focus group discussions) in northern Ghana.

**Results:**

Termites, crickets, grasshoppers, and caterpillars were recognized as the most prevalent edible insects in communities. These insects were largely consumed by children but presently only included in household meals by a few households. Individual, sociocultural, sensory characteristics of insects, climate, and economic aspects were cited as grounds for and against entomophagy. Existing community-based children’s diets were unable to meet the acceptable recommended nutrient intake (RNI; within a given age and gender group, the RNI is the amount of a nutrient ingested daily that would meet the needs of almost all healthy individuals in that group) level of all nutrients under consideration, especially for zinc, vitamin B-12, folate, and fat. Inclusion of community-based edible insects increased the RNI levels for all 11 micronutrients considered and met children’s zinc, vitamin B-12, folate, and fat requirements.

**Conclusions:**

Community-based insects demonstrate a great potential for meeting micronutrients needs of children in the research setting. Future research is required to improve households’ adoption of community-based insects as part of household meals and to make insects accessible to households.

## Introduction

Micronutrients deficiencies in children younger than 5 y continue to be worrisome globally, especially among countries with lower socioeconomic status [1,2]. Under-5 y of age is a critical period for children’s growth and development. Nutritional deficiency during this period is associated with wasting, underweight, and stunting, especially during the first 2 y of life [1]. The widespread of micronutrient deficiencies in children are due to poor diet quality and elevated needs of the subpopulation [[2], [3], [4]]. Strong evidence exists showing that diet quality is the key factor among a multitude contributing factors underlying micronutrient deficiencies [[5], [6], [7]].

In Ghana, undernutrition, specifically micronutrient deficiencies are a major problem among children [[8], [9], [10]]. Current evidence on nutrition indicators pinpoints northern Ghana as the most affected by undernutrition and micronutrient malnutrition. The Upper East region in northern Ghana has the greatest prevalence (29%) of stunting (chronic malnutrition), than a prevalence of 21% nationwide and 25% in northern Ghana [8]. Acute malnutrition, or wasting, has an average prevalence of 7% nationwide, which is significantly lower than the northern Ghana’s 11% prevalence [8]. Northern Ghana continues to have a high prevalence of micronutrient deficiencies [[8], [9], [10]], especially anemia. Comparing anemia and iron deficiencies among children younger than 5 y, anemia (53.2%) and iron deficiency anemia (29.0%) are more common in northern Ghana than those in southern Ghana at 32.3% for anemia and 5.2% for iron deficiency anemia [8].

An earlier study indicated that the requirements of some micronutrients (Fe, Zn, Ca, riboflavin, folate, and vitamins A, B-12, and C) were difficult to meet with available local foods and dietary patterns among children in northern Ghana [11,12]. These nutrients were referred to as “problem” nutrients. The finding on the problem nutrients further buttresses the assertion that inadequate quality of the diet ingested is indeed a major player driving the prevailing high prevalence of undernutrition and micronutrient deficiencies in northern Ghana. Recent data from Ghana’s Comprehensive Food Security and Vulnerability Analysis on diet quality indicate a significant prevalence of a nondiverse diet in northern Ghana [13] which supports this being a factor explaining the high prevalence of micronutrient deficiencies. Furthermore, the diets consumed are predominantly plant based [14,15], and the majority of the problem nutrients (especially zinc, vitamin B-12, iron, and folate) are found in more expensive animal source foods. The challenge is how to engage in diet modification that is affordable and acceptable to the local populations.

Considering that children’s diets are repetitive and mostly plant based [9,16] in northern Ghana, the beckoning and logical recommendation would be the inclusion of animal source foods to children’s diets. However, cost implications limit animal source food consumption among vulnerable populations [[17], [18], [19]]. An alternative and complementary route is entomophagy. Among the regions of Ghana, northern Ghana dominates in entomophagy [20]. Insects are a good source of many micronutrients and present an opportunity to improve food and nutrition security [21]. Anecdotal evidence, however, suggests that insects are often not used regularly in household meals and are not eaten by all household members. Moreover, despite their potential, little is known about insect consumption by children and the potential nutrient contribution of insects to a young child’s diet. In this light, this study aimed to *1*) document the kinds of commonly consumed insects and the reasons communities themselves give for or against entomophagy in the study area, *2*) document the reasons for the addition or nonaddition of insects to household meals, and *3*) determine the potential nutrient contribution of common insects within the community to a young child’s diet.

## Methods

### Study design and subjects

The study was conducted according to the guidelines of the Declaration of Helsinki and approved by the Committee on Human Research Publication and Ethics of University for Development Studies. Informed consent was obtained from all subjects involved in the study.

Both qualitative and quantitative research methods were concurrently applied in this exploratory study. First, 6 focus group discussions were conducted to gather information on the kinds of insects that are commonly eaten as well as reasons for and against entomophagy. We also explored the reasons for the addition or nonaddition of insects to household meals. Second, a semistructured questionnaire was administered to household meal preparers as they were expected to have the best knowledge of and practical experience regarding entomophagy at the household level. The questionnaire also gathered participants’ sociodemographic characteristics. Participants of the focus group discussions were a subset of the participants who responded to the semistructured questionnaire. Household food preparers were particularly recruited as they have in-depth knowledge of what is consumed at the household level as well as what young children eat in the research setting. Culturally, and per the study’s setting, household meal preparers are females.

Data were collected (November–December 2023) in 3 regions of Ghana: the Savannah Region (Central Gonja district—Kabilpe and Alipe communities), the North East Region (East Mamprusi district—Langbinsi and Bowku communities), and the Northern Region (Kumbungu district—Namdu and Limo communities). The selected districts and communities reflect ethnic and/or ecological diversity, as well as areas with poor socioeconomic level and high malnutrition rates. With the help of the OPTIFOOD software (version 4.0.5), this study used consumption data from an earlier study [12] to model the nutrient composition of food intake for children aged 1–3 y for 11 micronutrients, protein, and fat, with and without community-based insects.

### Nutrient adequacy of children’s foods

A nutrient-adequate diet is defined as the set of foods accessible at any given time and place that would keep dietary energy and all critical nutrients within lower and upper bounds. To calculate the nutrient-adequate diet for children, linear programming software (OPTIFOODs, version 4.0.9.0) was used to select food items from children's consumption that have nutrient content that meets the needs of 50% of the healthy population of children (aged 1–3 y), as well as the highest level of nutrients likely to prevent risk of adverse health effects [22], while specifying that the macronutrient intakes are within the acceptable macronutrient distribution range, and meeting the energy requirement of the age range. Children aged 1–3 y were of a particular interest owing to the higher prevalence of micronutrients deficiencies in this age brackets [8]. Additionally, the age bracket includes children who are breastfeeding and actively participate in household meals’ consumption.

The OPTIFOOD analysis provided recommendations that determined the quantity and frequency of consumption of available foods, expressed as the recommended number of servings per week. This analysis helps to address deficits in local diets and intake patterns. It also reveals whether such recommendations are insufficient to meet the nutrients under consideration’s requirements. Only foods reported to be consumed by children were used in this study to optimize nutrient intake adequacy. Following that, we included insects in the model to evaluate their potential to meet the needs of the identified problem nutrients. Detailed methods on analysis using the OPTIFOOD software have been published earlier [12]. Table 1 illustrates the general layout of our methodology for the modules for each scenario. The optimized diet with food groups indicates that whether consumption were to be based on available foods, limiting selection to the best nutrients source foods, with the caveat of having to obtain them from diverse reference food groups. Optimized diet without food groups, on the contrary, indicates consumption or selection of best nutrients source foods from available foods without considering the food groups that foods belong.

**TABLE 1:** OPTIFOOD analysis representation.

	Steps	Scenario
	Children’s foods	Children’s foods + insects
1	Module 1: screen diets for model parameters to ensure OPTIFOOD generates realistic diets	Module 1: screen diets for model parameters to ensure OPTIFOOD generates realistic diets
2	Module 2: identify draft recommendations for children’s foods only	Module 2: identify draft recommendations for children’s foods + common insects within the community
3	Module 3: test food-based recommendation for children’s foods only	Module 3: test food-based recommendation for children’s foods + common insects within the community

Nutrient intakes above 70% of recommended nutrient intake (RNI) were deemed acceptable. For most nutrients, this represents at least the estimated average requirement and enables comparability with previous studies [[23], [24], [25]]. Total fat, total protein, calcium, vitamin C, thiamin, riboflavin, niacin, vitamin B-6, folate, vitamin B-12, vitamin A, iron, and zinc are among the nutrients. Secondary data from a previous study [12] done in the Northern Region were used to construct models on children’s intake.

### Statistical analysis

#### Quantitative data

The estimated sample size (*n* = 392) was calculated using *N* = *t*^2^ × *p*(1 − *p*)/*m*^2^, the normal standard deviation (*t*) at a 95% confidence level, a margin of error (*m*) of 5%, 45.9% entomophagy prevalence (termite consumption) [20], and a 2.5% nonresponse rate. SAS (version 9.4) and OPTIFOOD software (version 4.0.9.0) were used to analyze the data. Using OPTIFOOD, the nutrient contribution of children's foods with and without insects was calculated and presented as % RNI.

##### Analysis in OPTIFOOD

All analyses were performed using a 2-tiered strategy to create weekly for the target food-based recommendations group using the OPTIFOOD software; the 2 scenarios that were modeled to develop food-based recommendations for children were a community-based children's diet without insects and a community-based children's diet with community-based insects. Our recent work [26] and earlier investigations [24,25,[27], [28], [29]] have outlined the usage of the OPTIFOOD software linear programming analyses used in this study.

#### Qualitative data

The focus group discussions were transcribed and analyzed using Excel spread sheets (Microsoft Excel, 2016) and color coding. Specific codes from the first phase were grouped into broader subcategories. To incorporate all of relationships between categories and subcategories into overarching themes, selective coding was used.

## Results

### Quantitative results

#### Sociodemographic characteristics and entomophagy information

The results in Table 2 indicate that nearly all the participants (80.8%) were married and had no formal education (80.0%). Most of the participants were Muslims (90.7%) and were mainly farmers (53.6%) of diverse ethnicity with majority being Dagombas (41.8%). Majority of the participants (77.9%) indicated that entomophagy was practiced in their respective communities and mostly among children (75.3%). Approximately one-fifth (24.1%) of the participants add insects to household meals. Table 3 indicates that commonly consumed community-based insects were termites, crickets, grasshoppers, and caterpillars. Beetles were mentioned as less consumed insects.

**TABLE 2:** Sociodemographic characteristics and insect consumption in participants’ communities.

Variable		Mean ± SD
Age		38.7 ± 11.8
Family Size		12.4 ± 7.7
		Count (% of category)
Ethnicity	Dagomba	163 (41.8)
	Gonja	85 (21.8)
	Manprusi	100 (25.6)
	Other	42 (10.8)
Religion	Christianity	35 (9.0)
	Islam	353 (90.7)
	Traditional	1 (0.3)
Sex	Male	3 (0.8)
	Female	385 (99.2)
Marital status	Single	6 (1.5)
	Married	315 (80.8)
	Separated	4 (1.0)
	Widowed	59 (15.1)
	Divorced	6 (1.5)
Educational level	Primary/JHS	54 (13.9)
	SHS	24 (6.2)
	None	312 (80.0)
Occupation	Farmer	208 (53.6)
	Trader	76 (19.6)
	Artisan	23 (5.9)
	Service industry	17 (4.4)
	Other	64 (16.5)
Consumption of insects in community	Yes	303 (77.9)
	No	22 (22.1)
Who consumes insects in the community	Mostly children	228 (75.3)
	Mostly women	6 (2.0)
	Mostly adults	21 (6.9)
	Every one	48 (15.8)
Add insects to household meals	Yes	73 (24.1)
	No	230 (75.9)

Abbreviations: JHS, junior higher secondary; SD, standard deviation; SHS, senior higher secondary.

**TABLE 3:** Commonly consumed insects in northern Ghana.

District	Community	Insects consumed				
		Caterpillar	Cricket	Termite	Grasshopper	Beetle
Central Gonja	Alipe		XX	XX	X	X
	Kabilpe		XX	XX		
East Manprusi	Langbinsi	XX	XX	XX	XX	
	Bowku	XX	XX	XX	XX	
Kumbungu	Namdu		XX	XX		X
	Limo		XX	XX		X

#### Nutrients contribution of community-based insects

Table 4 indicates the nutrient contribution of children’s foods with and without insects. The nutrient contribution (%RNI) of children’s foods for seven 7-d diet simulations for the 2 scenarios. The food pattern diet (children’s foods without insects) had 4 and 8 nutrients with %RNI of ≥70 for optimized diet with food groups and without food groups, respectively. For the no-restriction diet modeled in scenario 2, the %RNI for children’s food improved tremendously. Using children’s foods without insects as a base, 10 and 12 nutrients with %RNI of ≥70 were observed for the addition of community-based insects for optimized diet with food groups and without food groups, respectively. The model with insects suggests that zinc, vitamin B12, folate, and fat requirements were met with the addition of insects in children’s diet in northern Ghana.

**TABLE 4:** Optimized diets of non–breastfeeding children (1–3 y) using their usual consumption with and without insects in northern Ghana.

Analysis of children’s diets	Protein (%)	Fat (%)	Ca (%)	Vit C (%)	Vit B1 (%)	Vit B2 (%)	Vit B3 (%)	Vit B6 (%)	Vit B9 (%)	Vit B12 (%)	Vit A (%)	Fe (%)	Zn (%)
Diet with Food Group + insects	437	67.9	31.9	81.4	105.5	247.5	237.2	8237.3	73.2	145.9	37.3	578.6	110.6
Diet without Food Group + insects	445.9	81.8	49.2	119.2	100	280.2	243.6	8249	87.8	168.8	93.5	611	118.5
Diet with Food Group	201.2	55	18.2	35.4	120.2	47.7	98.4	138	43.2	6.7	25.9	52.2	39
Diet without Food Group	220.8	65.4	33.6	72.4	126.7	80.9	100	155.6	55.6	29.3	93.6	85.4	47.7

### Qualitative results

Table 5 provides insights to the reasons for the consumption and nonconsumption of insects in some communities in northern Ghana regardless of whether they are consumed as snacks or as part of a formal meal. Table 6 focuses on the reasons for addition and exclusion of insects in household meals.

**TABLE 5:** Reasons for consumption or nonconsumption of insects in northern Ghana.

Study question	Theme	Exemplars
Why do people eat insects in your community?	Culturally acceptable	“Crickets and termites have been part of our traditional foods for ages” “Our ancestors ate these insects and the tradition has been passed on to us”
	Cheap alternative animal source food	“Insects are cheaper alternative or replacement for fish and meat” “We eat them because they are cheaper to obtain”
	Nutritional and health benefits	“We eat them because they help our bodies to grow well” “Eating insects make you healthy and strong”
	Taste preference	“We eat them because of their taste, termites especially are very tasty” “Some insects such as termites and crickets are very delicious”
	Satiety	“When you eat termites, you feel full the whole day, you drink a lot of water throughout the day” “When you eat termites, you feel full easily”
Why do people not eat insects in your community?	Nauseating effects	“Some people vomit when they eat them” “They look dirty and make some people to vomit”
	Limited knowledge on insects	“Some people have no knowledge on insects as food” “Others don’t know how to prepare insects to make them tasty and edible”
	Personal preference	“Individuals choose what to eat, some people just don’t like insects” “People don’t eat them for personal reasons”

**TABLE 6:** Reasons for addition and exclusion of insects in household meals.

Study question	Theme	Exemplars
Why are insects added to household meals?	Taste preference	“Adding insects to meals make them tastier” “Some insects such as termites make soup delicious” “Caterpillars have no competitor in enhancing soup especially aleefu soup”
	Nutritional and health benefits	“Eating insects make you healthy and strong” “Insects in meals provide important elements that the body needs to function well.”
	Cheap alternative for animal source food	“Insects are cheaper alternative or replacement for fish whenever they are in season” “We use them because they are cheaper to get when in season”
	Cultural acceptance	“As kids we saw our parents add them to soups so we grew up doing same but now the insects are difficult to come by” “Insects as food have been part our lives”
	Personal preference	““I don't know why people add insects to household meals, reasons are best known by individuals or households that add them” “I can’t really tell why some community members add them to soups, the individuals involved can best explain”
Why are insects not added to household meals?	Inferior alternative	“When you have fish why will you be cooking insects, they are not as good as amani” “Insects do not taste as good as meat and fish. We don’t eat them because we have better alternatives such as fish and other meats”
	Inaccessibility of insects as food	“Termites are now scarce, they are no longer plentiful as they used to be, we don’t get enough to add to soups any longer” “…the quantities we get are not enough for the kids and then for household soups”
	Inappropriate for household meals	“When you add insects to soup, they will float on the soup. They are not really good for soups” “We prefer to eat insects by chewing them than adding to soup. They are not appropriate for soup”
	Varying household members food preferences	“They are not added to soups because not everyone in the household can eat them in soup” “Insects are not added to household meals because some people don’t like them and they don’t eat them”
	Known as children’s food	“Insects are meant for children and not grown-ups so they are not added to soups” “Insects are not meant for grown-ups”
	Bad smell	“Most of these insects do not have good smell, they will spoil household meals” “Some people choose not to add insects to household meals because of their bad smell”
	Personal preference	“I don't know, we don’t taboo them, those who don’t add insects have their personal reasons” “As for me, I cannot tell why people don’t add insects to household meals, maybe the people who don’t add them will be in the position to give their reasons”

In Table 5, emergent themes for consumption of insects included cultural acceptance, insects being cheaper alternative for animal source food, satiety, taste preference for insects, nutritional and health benefits derived from insects. For nonconsumption, emergent themes included limited knowledge on insects, nauseating effects, and personal preference. Exemplars of participants’ responses under the respective themes are indicated further.

### Reasons for entomophagy

#### Cultural acceptance

Respondents indicated that insects have been part of their traditional diets, and this has been passed down to generations. For example,

“Our ancestors ate these insects and the tradition has been passed on to us.”

#### Cheap alternative for animal source food

Some respondents indicated that insects are replacements or substitutes for meat and fish, which are expensive and not readily accessible:

“Insects are cheaper alternative or replacement for fish and meat.”

#### Taste preference

The tasty nature of some insects is the reason why some community members consume them:

“We eat them because of their taste, termites especially are very tasty.”

#### Nutritional and health benefit

Nutritional and health benefits derived from insects’ consumption inform the reason for their consumption among some community members. For example,

“We eat them because they help our bodies to grow well.”

#### Satiety

The satiety nature of insects did inform why some community members engaged in entomophagy:

“When you eat termites, you feel full easily.”

### Reasons against entomophagy

#### Nauseating effects

Respondents indicated that insects trigger nauseating effects, making some people to vomit upon consuming them:

“Some people vomit when they eat them.”

#### Limited knowledge on insects

Respondents are also of the view that some community members do not eat insects due to limited knowledge on insects as food:

“…..some people have no knowledge on insects as food.”

#### Personal preference

As with food choices and food habits, personal preference plays a key role as to what to eat or not to eat:

“Individuals choose what to eat, some people just don’t like insects.”

Detail results on the reasons for the inclusion or noninclusion of insects in household meals are presented in Table 6. With the exception of satiety, emergent themes for the inclusion of insects in household meals (cultural acceptance, cheaper alternative for animal source food, taste preference, nutritional and health benefits) are same as themes for communities’ consumption of insects. For noninclusion, emergent themes included inferior alternative, inaccessibility of insects as food, inappropriate for household meals, varying household members’ food preferences, known as children’s food, bad smell, and personal preference. Exemplars of participants’ responses under the respective themes are provided further.

### Reasons for inclusion of insects in household meals

#### Cultural acceptance

Respondents indicated that insects have been part of their traditional diets and this has been passed down to generations:

“As kids we saw our parents add them to soups so we grew up doing same but now the insects are difficult to come by.”

#### Cheap alternative for animal source food

Some respondents indicated that insects are substitutes for meat and fish, with the latter being expensive and not readily accessible:

“Insects are cheaper alternative or replacement for fish whenever they are in season.”

#### Taste preference

The tasty nature of some insects is the reason why they are added to household meals:

“Some insects such as termites make soup delicious.”

#### Nutritional and health benefits

Insects are also being added to household meals for their nutritional and health benefits:

“Eating insects make you healthy and strong.”

### Reasons for noninclusion of insects in household meals

#### Inferior alternative

Participants are of the view that insects are inferior substitutes for meat and fish. Once they have access to superior products, they no longer go in for inferior alternatives:

“When you have fish why will you be cooking insects, they are not as good as amani (dried anchovies).”

#### Inaccessibility of insects as food

Some participants indicated that they no longer add insects to household meals as insects are inaccessible nowadays:

“Termites are now scarce, they are no longer plentiful as they used to be, we don’t get enough to add to soups any longer.”

#### Inappropriate for household meals

Adding insects to household meals especially soups is deemed inappropriate by some participants. Apparently, for these nonconsumers, a major reason is the texture of the insects. For this reason, they choose not to add insects to household meals:

“We prefer to eat insects by chewing them than adding to soup. They are not appropriate for soup.”

#### Varying household members’ food preferences

Household meals are served to a number of people with varying preferences. In some cases, some household members do not like or consume insects, thus hindering their inclusion in household meals:

“Insects are not added to household meals because some people don’t like them and they don’t eat them.”

#### Known as children’s food

Insects are considered as children’s food by some participants as children often harvest them:

“Insects are not meant for grown-ups.”

#### Bad smell

The sensory attributes also informed the inclusion of insects in household meals or otherwise. Some participants indicated that insects have bad smell, so they do not add them to household meals:

“Some people choose not to add insects to household meals because of their bad smell.”

#### Personal preference

Individual differences play a crucial role as to what they choose to eat or not to eat. Participants indicated that noninclusion of insects in household meals is based on personal preferences:

“I don't know, we don’t taboo them, those who don’t add insects have their personal reasons.”

## Discussion

Available local foods and existing dietary patterns among children in northern Ghana are insufficient to meet their micronutrient requirements [11,12], owing to children’s nondiverse and predominantly plant-based diets [9,16]. Aside from enhancing diets with animal-sourced foods, which is limited by cost [[17], [18], [19]], another option is entomophagy. This study investigated and documented the types of insects that are commonly consumed, as well as the reasons for or against entomophagy. In addition, determine the nutrient contribution of edible community-based insects in children’s diets was assessed.

### Entomophagy, reasons for or against its practice

The results of our study suggest that termites, crickets, grasshoppers, and caterpillars were the commonly consumed insects in the study areas, which is in consonance with an earlier study in Nigeria and other sub-Saharan African countries [[30], [31], [32]]. Entomophagy is practiced in all districts in our study area. The qualitative results are further corroborated by the quantitative results in which 78% of the respondents confirmed the practice of entomophagy in their respective communities.

#### Reasons for entomophagy

The reasons for entomophagy in the communities and their inclusion in household meals were diverse. Cultural acceptance, insects being cheaper alternative for animal source food, taste preference for insects, and nutritional and health benefits derived from insects were the key reasons for entomophagy at community level as well as in household meals. These findings are in agreement with an earlier study, which indicated that nutritional value, sensory attributes, and tradition/culture positively influenced entomophagy [33]. A cross-cultural qualitative study in Thailand and Netherlands also echoed the positive role of cultural acceptance of insects as food in the practice of entomophagy [34]. A Nigerian study also indicated that insects being cheaper alternative for animal source food, taste preference for insects, and nutritional and health benefits derived from insects were factors that influenced entomophagy [30].

The satiety derived from insect ingestion was also reported at the community level as one of the reasons for practicing entomophagy. Confirmation of the satiating potential of insects was illustrated by an earlier study, which indicated that insects increased the satiating potential of foods [35]. Additionally, another study indicated that the ability of insect-derived protein from crickets to stimulate postprandial hyperaminoacidemia and regulate satiety is much effective than conventional animal-derived proteins [36]. These findings corroborate our finding on satiety at the community level.

#### Reasons against entomophagy

In contrast, the reasons for nonconsumption of insects differ depending on the community or household level interaction. Personal preference for nonconsumption of insects was the only shared reason for not practicing entomophagy per community-level and household-level engagements. Decision on what to eat is complex and vary per individuals [37]. It was not surprising that personal preference emerged as a significant theme in both levels. An earlier study intimated that personal preference, experience, and/or familiarity with insects influenced consumption or otherwise of insects [33]. Leng et al. [38] corroborated these findings by indicating that for food choices, among other factors, personality characteristics influence individuals’ choices [38].

Limited knowledge on insects as food and nauseating effects of insects emerged at the community-level engagement as reasons for nonconsumption of insects. These findings are in agreement with an earlier study, which suggested that unawareness of insects as food negatively influenced entomophagy in Nigeria [30]. Nauseating and vomiting on consuming insects were also reported in studies in Thailand [39] and Reunion Island [40]. This unpleasant experience may deter its victims from entomophagy.

At the household level, for noninclusion of insects in household meals, key reasons included the following: insects being considered as inferior alternative, inaccessibility or unavailability of insects as food, inappropriateness of insects for household meals, varying household members’ food preferences, insects being reserved as children’s food, and bad smell from insects. Our findings on the inaccessibility or unavailability of insects as food, bad smell from insects, and inappropriateness of insects for household meals (which is largely on texture) are in agreement with those of other studies that suggested that unavailability of insects [30] and sensory attributes (tastes, texture, and smell) [33] negatively influenced entomophagy.

Insects being considered as children’s food came out strongly in the focus group discussions as well as in the semistructured questionnaire’s results. It is not therefore surprising that this belief negatively influenced the inclusion of insects in household meals. An earlier study indicated that community’s beliefs about what its members should eat determines their choices of food [41]. In another study, Chakona [42] indicated that social circumstance such as beliefs dictate its members’ feeding practices. These findings explain why the communities’ belief (insects are children’s food) negatively influenced entomophagy in household meals.

Household meals are meant for a number of people, as such, preferences of household members, especially key adult decision makers inform what goes into the cooking pot. Varying household members’ food preferences concerning entomophagy in household meals is only natural, especially when such foods are seen as *“*children’s food*”.* The influence of this emergent theme is also observed in the quantitative results; only one-fifth of participants add insects to household meals. A study in Nigeria is also in agreement with our finding on varying household members’ food preferences. The stated study indicated that influence of family members dictates the use of insects as food [30]. Additionally, some participants indicated that insects were inferior alternative for meat, this might also influence the inclusion of insects in household meals.

### Nutrient contribution of community-based insects to children’s food

The study findings revealed that existing children’s diets were unable to meet the recommended RNI levels for all nutrients under consideration. According to previous research [11,12], meeting the requirements for some micronutrients (Fe, Zn, Ca, riboflavin, folate, and vitamins A, B-12, and C) was problematic. It was also nearly impossible to achieve zinc, vitamin B-12, folate, and fat requirements for children on current diets, regardless of dietary combinations. These findings are not surprising, given that Ghana’s Comprehensive Food Security and Vulnerability Analysis research [13] revealed children’s poor dietary quality. Earlier studies in Ghana also suggested that existing children’s diets were inadequate to meet their nutrient requirements [14,15]. Finding a solution to meet the inadequacy in children’s diet is thus urgently needed.

The addition of edible community-based insects to children’s diets increased the RNI level for all nutrients under study. With the exception of calcium, it was possible to achieve the requirements for all nutrients under consideration by the inclusion of community-based edible insects in children’s diets. The nutrient quality of insects is comparable with that of meat, and in certain cases, it exceeds that of meat [43]; so, it is not surprising that insects have the ability to improve the quality of children’s diets. Although some studies consider insects a threat to food security [44,45], others believe insects hold the key to global food and nutrition security, particularly in underdeveloped nations [[46], [47], [48]]. Given the high-quality protein and micronutrients, possible environmental and economic benefits of edible insects [46], and the existing challenges in our study location, we side with the latter.

### Limitations

We understand that there are several substantial limitations to our study. First, rather than assessing the amount of food currently consumed by children, we used secondary data. Furthermore, the RNIs were calculated using nutritional composition tables, which may not completely account for nutrient bioavailability within a given food matrix. As a result, although we are confident that the proportion of nutrients reaching the cutoff points was met in the majority of cases, there is likely to be some uncertainty about these anticipated values owing to the lack of bioavailability adjustment. In addition, seasonal availability of the insects may limit their potential to contribute to the diet at selected times of year. This needs further investigation.

In conclusion, entomophagy is culturally accepted and practiced in all communities in our study area. Common community-based edible insects included termites, crickets, grasshoppers, and caterpillars. These insects were mostly eaten by children and added to household meals by few households. Diverse reasons were given for and against entomophagy. Existing community-based children’s foods were unable to meet the desirable RNI level of all nutrients under consideration and it was practically impossible to meet zinc, vitamin B-12, folate, and fat requirements. Inclusion of community-based edible insects improved RNI level of all nutrients and met zinc, vitamin B-12, folate, and fat requirements for children.

## Acknowledgments

We appreciate and acknowledge the support of Cabral.V. Bantiu during the OPTIFOOD analysis. For making the secondary data accessible to us, we are grateful to Global Alliance for Improved Nutrition (GAIN) and N2Africa: Putting Nitrogen fixation to work for smallholder farmers in Africa (www.N2Africa.org). We are equally thankful to Fauta Azupogo and Ilse de Jager for making the prepared OPTIFOOD input files accessible.

### Authors contributions

The authors’ responsibilities were as follows – CKK: conceptualized the study, guided the data collection and analyses, and wrote the draft manuscript; MB collected the primary data and reviewed the manuscript; JL: reviewed the manuscript and made substantial inputs; and all authors: have read and agreed to the published version of the manuscript.

## Conflict of interest

The authors report no conflict of interest.

## Funding

The authors reported no funding received for this study.

## Data availability

The data that support the findings of this study are available from the corresponding author upon request.
